# Supplementary material for: Hematological Ratios Are Associated with Acute Kidney Injury and Mortality in Patients That Present with Suspected Infection at the Emergency Department
Source: J Clin Med. 2022 Feb 16;11(4):1017. doi: 10.3390/jcm11041017 (PMC8874958; doi:10.3390/jcm11041017)
Supplement: Supplementary file 1 [file jcm-11-01017-s001.zip › jcm-1530343-supplementary.pdf]

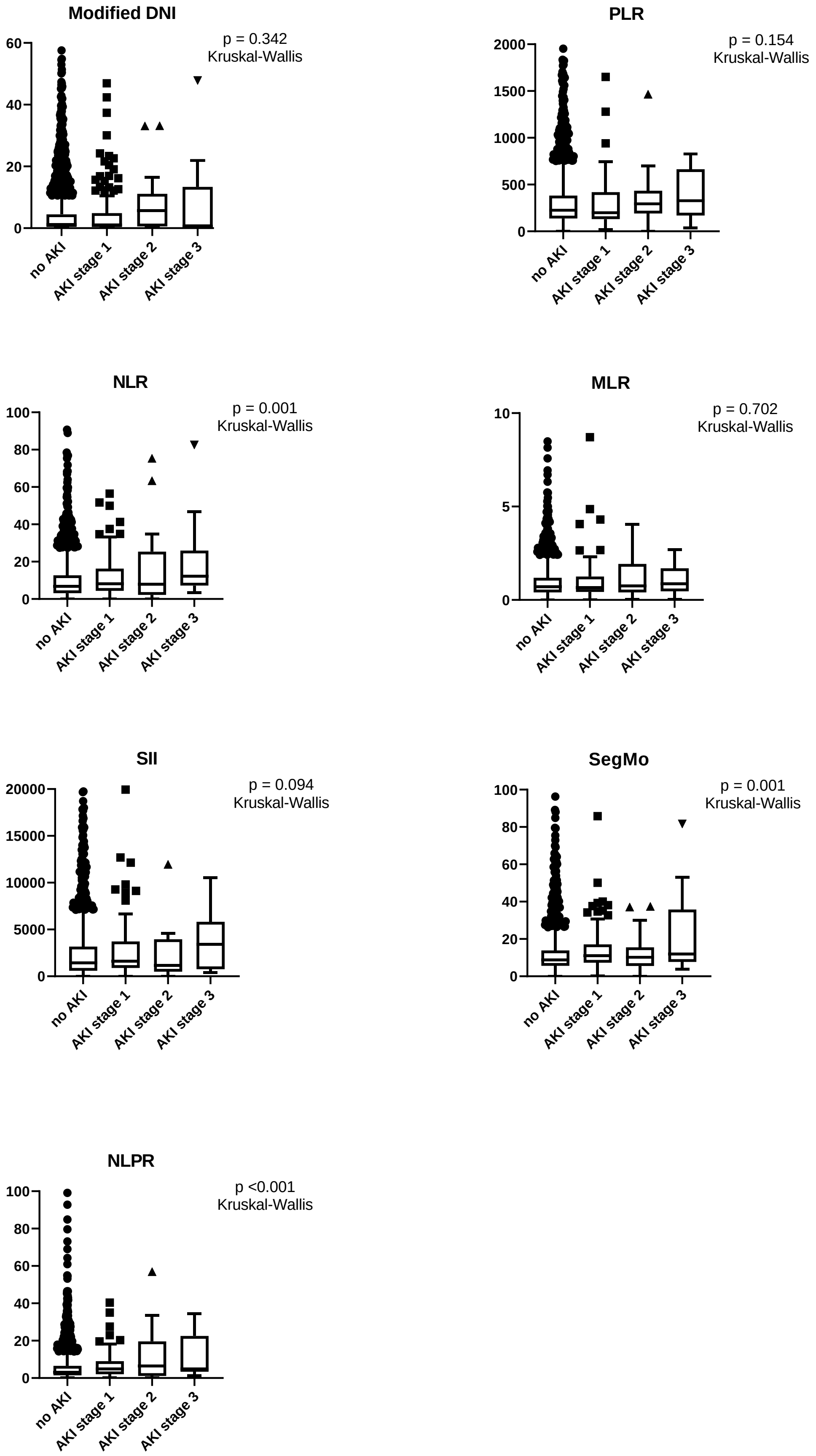

Figure S1. Distribution of hematological ratios after stratification for AKI stage.

Table S1. HRs (95% CI) for AKI <14 days after ED presentation.

	**Univariate**	**Multivariate**			
**Ratios**	**Crude HR (95% CI)**	**Adjusted HR: Model 1[a] (95% CI)**	**Adjusted HR: Model 2[b] (95% CI)**	**Adjusted HR: Model 3[c] (95% CI)**	**Adjusted HR: Model 4[d] (95% CI)**
**Modified DNI**					
Tertile 1, ≤ 0.5931	1.0 (reference)	1.0 (reference)	1.0 (reference)	1.0 (reference)	1.0 (reference)
Tertile 2, 0.5931 - 2.7088	**0.6 (0.4 - 0.9)**	**0.6 (0.4 - 1.0)**	**0.6 (0.4 - 1.0)**	**0.6 (0.4 - 1.0)**	**0.6 (0.4 - 1.0)**
Tertile 3, > 2.7088	0.9 (0.6 - 1.4)	0.9 (0.6 - 1.4)	0.9 (0.6 - 1.3)	0.9 (0.6 - 1.3)	0.8 (0.6 - 1.3)
**NLR**					
Tertile 1, ≤4.2805	1.0 (reference)	1.0 (reference)	1.0 (reference)	1.0 (reference)	1.0 (reference)
Tertile 2, 4.2805 - 10.2276	**1.7 (1.1 - 2.8)**	**1.7 (1.0 - 2.7)**	**1.7 (1.0 - 2.7)**	1.6 (1.0 - 2.7)	1.6 (0.9 - 2.6)
Tertile 3, >10.2276	**2.6 (1.6 - 4.1)**	**2.4 (1.5 - 3.9)**	**2.3 (1.4 - 3.7)**	**2.3 (1.4 - 3.6)**	**2.1 (1.3 - 3.5)**
**MLR**					
Tertile 1, ≤0.5057	1.0 (reference)	1.0 (reference)	1.0 (reference)	1.0 (reference)	1.0 (reference)
Tertile 2, 0.5057 - 0.9830	1.2 (0.8 - 1.8)	1.1 (0.7 - 1.7)	1.1 (0.7 -1.7)	1.1 (0.7 - 1.7)	1.0 (0.7 -1.6)
Tertile 3, >0.9830	1.2 (0.8 - 1.9)	1.1 (0.7 - 1.7)	1.1 (0.7 - 1.8)	1.1 (0.7 - 1.7)	1.1 (0.7 - 1.7)
**SMR**					
Tertile 1, ≤ 6.7500	1.0 (reference)	1.0 (reference)	1.0 (reference)	1.0 (reference)	1.0 (reference)
Tertile 2, 6.7500 -11.9633	**1.7 (1.0 - 2.7)**	1.6 (1.0 - 2.6)	1.6 (1.0 - 2.6)	1.6 (1.0 - 2.6)	1.5 (0.9 - 2.4)
Tertile 3, >11.9633	**2.4 (1.5 - 3.9)**	**2.4 (1.5 - 3.8)**	**2.3 (1.4 - 3.7)**	**2.2 (1.4 - 3.6)**	**2.1 (1.3 - 3.4)**
**PLR**					
Tertile 1, ≤161.6468	1.0 (reference)	1.0 (reference)	1.0 (reference)	1.0 (reference)	1.0 (reference)
Tertile 2, 161.6468 - 314.6356	0.9 (0.6 - 1.5)	0.9 (0.6 - 1.4)	0.9 (0.6 - 1.4)	0.9 (0.6 - 1.4)	0.9 (0.6 - 1.4)
Tertile 3, 314.6356	1.1 (0.7 - 1.7)	1.1 (0.7 - 1.6)	1.0 (0.7 - 1.6)	1.0 (0.7 - 1.6)	1.0 (0.7 - 1.5)
**NLPR**					
Tertile 1, ≤1.9151	1.0 (reference)	1.0 (reference)	1.0 (reference)	1.0 (reference)	1.0 (reference)
Tertile 2, 1.9151 - 5.0605	1.5 (0.9 - 2.4)	1.4 (0.8 - 2.3)	1.4 (0.8 - 2.3)	1.4 (0.8 - 2.3)	1.3 (0.8 - 2.2)
Tertile 3, >5.0605	**2.8 (1.8 - 4.4)**	**2.6 (1.6 - 4.1)**	**2.5 (1.6 - 4.0)**	**2.5 (1.6 - 4.0)**	**2.4 (1.5 - 3.8)**
**SII-index**					
Tertile 1, ≤869.43	1.0 (reference)	1.0 (reference)	1.0 (reference)	1.0 (reference)	1.0 (reference)
Tertile 2, 869.43 - 2414.46	1.6 (1.0 - 2.6)	1.6 (1.0 - 2.5)	1.6 (1.0 - 2.6)	1.5 (1.0 - 2.5)	1.5 (0.9 - 2.4)
Tertile 3, > 2414.46	**1.8 (1.1 - 2.8)**	**1.7 (1.1 - 2.8)**	**1.7 (1.1 - 2.7)**	**1.6 (1.0 - 2.6)**	1.6 (1.0 - 2.5)

Abbreviations: HR, Hazard Ratio; AKI, acute kidney injury; DNI, Delta neutrophil index; NLR, Neutrophil-to-lymphocyte ratio; MLR, Monocyte-to-lymphocyte ratio; SMR, Segmented neutrophil-to-monocyte ratio; PLR, Platelet-to-lymphocyte ratio; NLPR, Neutrophil-to-lymphocyte-platelet ratio; SII, Systemic immune-inflammation index

a. Correction made for: age, gender.
b. Correction made for: age, gender, comorbidity score, baseline renal function, immune status.
c. Correction made for: age, gender, comorbidity score, baseline renal function, immune status, medication use.
d. Correction made for: age, gender, comorbidity score, baseline renal function, immune status, medication use, disease severity, provisional diagnosis in the emergency department.

Table S2. HR's (95% CIs) for 14-day all-cause mortality.

	Univariate	Multivariate			
**Ratios**	**Crude HR (95% CI)**	**Adjusted HR: Model 1[a] (95% CI)**	**Adjusted HR: Model 2[b] (95% CI)**	**Adjusted HR: Model 3[c] (95% CI)**	**Adjusted HR: Model 4[d] (95% CI)**
**Modified DNI**					
Tertile 1, ≤ 0.5931	1.0 (reference)	1.0 (reference)	1.0 (reference)	1.0 (reference)	1.0 (reference)
Tertile 2, 0.5931 - 2.7088	**0.2 (0.1 - 0.6)**	**0.3 (0.1 - 0.7)**	**0.2 (0.1 - 0.7)**	**0.2 (0.1 - 0.6)**	**0.3 (0.1 - 0.7)**
Tertile 3, > 2.7088	1.0 (0.5 - 1.8)	1.0 (0.6 - 1.9)	1.1 (0.6 - 2.0)	1.1 (0.6 - 2.0)	1.0 (0.5 - 1.9)
**NLR**					
Tertile 1, ≤4.2805	1.0 (reference)	1.0 (reference)	1.0 (reference)	1.0 (reference)	1.0 (reference)
Tertile 2, 4.2805 - 10.2276	1.2 (0.5 - 2.7)	1.1 (0.5 - 2.4)	1.0 (0.5 - 2.4)	1.1 (0.5 - 2.5)	1.1 (0.5 - 2.4)
Tertile 3, >10.2276	**2.1 (1.0 - 4.3)**	1.8 (0.9 - 3.7)	1.8 (0.9 - 3.8)	2.0 (1.0 - 4.1)	1.8 (0.8 - 3.7)
**MLR**					
Tertile 1, ≤0.5057	1.0 (reference)	1.0 (reference)	1.0 (reference)	1.0 (reference)	1.0 (reference)
Tertile 2, 0.5057 - 0.9830	0.7 (0.3 - 1.5)	0.6 (0.3 - 1.3)	0.6 (0.3 - 1.3)	0.6 (0.3 - 1.3)	0.5 (0.2 - 1.2)
Tertile 3, >0.9830	1.3 (0.7 - 2.4)	1.1 (0.6 - 2.1)	1.1 (0.6 - 2.1)	1.2 (0.6 - 2.2)	1.0 (0.5 - 2.0)
**SMR**					
Tertile 1, ≤ 6.7500	1.0 (reference)	1.0 (reference)	1.0 (reference)	1.0 (reference)	1.0 (reference)
Tertile 2, 6.7500 -11.9633	2.1 (0.9 - 4.9)	1.9 (0.8 - 4.5)	1.9 (0.8 - 4.3)	1.8 (0.8 - 4.3)	1.8 (0.8 - 4.3)
Tertile 3, >11.9633	**2.7 (1.2 - 6.3)**	**2.6 (1.2 - 5.9)**	**2.6 (1.2 - 5.9)**	**2.7 (1.2 - 6.2)**	**2.3 (1.0 - 5.4)**
**PLR**					
Tertile 1, ≤161.6468	1.0 (reference)	1.0 (reference)	1.0 (reference)	1.0 (reference)	1.0 (reference)
Tertile 2, 161.6468 - 314.6356	1.0 (0.4 - 2.3)	0,9 (0.4 - 2.2)	1.0 (0.4 - 2.2)	1.0 (0.4 - 2.3)	1.1 (0.5 - 2.5)
Tertile 3, 314.6356	**2.3 (1.1 - 4.7)**	**2.1 (1.0 - 4.2)**	2.0 (1.0 - 4.0)	2.0 (1.0 - 4.1)	1.9 (0.9 - 3.9)
**NLPR**					
Tertile 1, ≤1.9151	1.0 (reference)	1.0 (reference)	1.0 (reference)	1.0 (reference)	1.0 (reference)
Tertile 2, 1.9151 - 5.0605	**2.9 (1.2 - 6.8)**	**2.6 (1.1- 6.2)**	**2.6 (1.1 - 6.3)**	**2.9 (1.2 - 6.8)**	**2.6 (1.1 - 6.3)**
Tertile 3, >5.0605	**2.9 (1.2 - 6.8)**	**2.5 (1.0 - 5.9)**	**2.7 (1.1 - 6.4)**	**3.0 (1.2 - 7.1)**	**2.6 (1.1 - 6.3)**
**SII-index**					
Tertile 1, ≤869.43	1.0 (reference)	1.0 (reference)	1.0 (reference)	1.0 (reference)	1.0 (reference)
Tertile 2, 869.43 - 2414.46	0.5 (0.2 - 1.2)	0.5 (0.2 - 1.1)	0.5 (0.2 - 1.1)	0.5 (0.2 - 1.2)	0.5 (0.2 - 1.2)
Tertile 3, > 2414.46	1.9 (1.0 - 3.6)	1.7 (0.9 - 3.2)	1.6 (0.8 - 3.1)	1.7 (0.9 - 3.3)	1.5 (0.8 - 2.9)

Abbreviations: HR, Hazard Ratio; AKI, acute kidney injury; DNI, Delta neutrophil index; NLR, Neutrophil-to-lymphocyte ratio; MLR, Monocyte-to-lymphocyte ratio; SMR, Segmented neutrophil-to-monocyte ratio; PLR, Platelet-to-lymphocyte ratio; NLPR, Neutrophil-to-lymphocyte-platelet ratio; SII, Systemic immune-inflammation index

a. Correction made for: age, gender.
b. Correction made for: age, gender, comorbidity score, baseline renal function, immune status.
c. Correction made for: age, gender, comorbidity score, baseline renal function, immune status, medication use.
d. Correction made for: age, gender, comorbidity score, baseline renal function, immune status, medication use, disease severity, provisional diagnosis in the emergency department.
